# Preparation of Lignan-Rich Extract from the Aerial Parts of *Phyllanthus niruri* Using Nonconventional Methods

**DOI:** 10.3390/molecules25051179

**Published:** 2020-03-05

**Authors:** Meselhy R. Meselhy, Ola E. Abdel-Sattar, Sahar El-Mekkawy, Ahmed M. EL-Desoky, Shanaz O. Mohamed, Sobhy M. Mohsen, Essam Abdel-Sattar, Ali El-Halawany

**Affiliations:** 1 Department of Pharmacognosy, Faculty of Pharmacy, Cairo University, Kasr El Aini st., Cairo 11562, Egypt; meselhy.meselhy@pharma.cu.edu.eg (M.R.M.); ola.abdelsattar@gmail.com (O.E.A.-S.); essam.abdelsattar@pharma.cu.edu.eg (E.A.-S.); 2 Department of Chemistry of Natural Compounds, National Research Centre, Dokki 12622, Egypt; saheg.2011@gmail.com; 3 Department of Molecular Biology, Genetic Engineering and Biotechnology Research Institute (GEBRI), University of Sadat City (USC), Sadat City 32958, Egypt; ahmed.desoky334@gmail.com; 4 School of Pharmaceutical Sciences, Universiti Sains Malaysia, Gelugor, Penang 11700, Malaysia; shahnas@mynaturalwellness.com; 5 Department of Food Science and Technology, Faculty of Agriculture, Cairo University, Giza 12613, Egypt; sobmohsen1@hotmail.com

**Keywords:** *Phyllanthus niruri* L., lignans, phyllanthin, nonconventional methods, alkaline digestion, microwave, enzyme-assisted extraction

## Abstract

Phyllanthin and related lignans were found to be responsible, at least in part, for most of the activity of *Phyllanthus* species. This observation encouraged the authors to develop methods for the preparation of an extract rich in phyllanthin and related lignans from the aerial parts of *P. niruri* L. Direct extraction with solvents produced extracts with variable yields and contents of lignans. Lignans were identified by LC-ESI-MS analysis as phyllanthin (used as marker substance), hypophyllanthin, phylltetralin, nirtetralin, and niranthin. Extraction with boiling water produced 18.10 g% (*w*/*w*) extract with a trace amount of lignans (phyllanthin content of 0.33 ± 0.10 mg/g extract), while extraction with MeOH gave 3.6 g% *w*/*w* extract with a low phyllanthin content (3.1 mg/g extract), as determined by HPLC. However, Soxhlet extraction with hexane, CH_2_Cl_2_, or acetone gave extracts with low yields (0.82, 1.12, and 3.40 g% *w*/*w*, respectively) and a higher phyllanthin contents (36.2 ± 2.6, 11.7 ± 1.68, and 11.7 ± 1.10 mg/g extract, respectively). Extraction quality and efficiency were optimized by adopting the following three different approaches: (1) Alkaline digestion of the plant material with 30% potassium hydroxide yielded 3.1 g% *w*/*w* of purified extract with high phyllanthin content (22.34 ± 0.13 mg/g); (2) microwave-assisted extraction using 80% MeOH gave an extract with a better yield (8.13 g% *w*/*w*) and phyllanthin content (21.2 ± 1.30 mg/g) (after filtration through a Diaion HP-20 column); and (3) treatment of the ground plant material at 50 °C with two hydrolytic enzymes, cellulase (9 U/g for 12 h) and then, protease (4 U/g up to 72 h) optimized the yield of extract (13.92 g% *w*/*w*) and phyllanthin content (25.9 mg/g extract and total lignans content of 85.87 mg/g extract). In conclusion, the nonconventional methods presented here are superior for optimizing the yield of extract and its lignan contents from the aerial parts of *P. niruri*.

## 1. Introduction

*Phyllanthus niruri* L. (family Phyllanthaceae) is a small herb with a wide range of medicinal properties. The whole plant has a long history of being used as a remedy for kidney stones, gallbladder stones, and liver-related diseases [1,2]. In addition, it is used as one of the components of a multiherbal preparation for treating liver ailments [2], and shows anti-inflammatory, antitumor, antinociceptive, and antioxidant properties. The spray-dried aqueous extract of the aerial parts of *P. niruri* was found to selectively inhibit the proliferation of cancer cell lines; human hepatocellular carcinoma cells (HepG2 and Huh-7) and colorectal carcinoma cells (Ht29), but was protective towards the normal cells, keratinocytes (HaCaT) [3]. In addition, the aqueous extract demonstrated both radical scavenging and hepatoprotective activities [4]. The 50% methanolic extract demonstrated hepatoprotective effect against nonalcoholic fatty liver disease (NAFLD) progression in rats, as well as reduced visceral adiposity, improved liver enzymes abnormalities, and decreased hepatic lipid peroxidation and fat accumulation. Both phyllanthin and gallic acid were found to be the main constituents in the extract [5]. Both the methanolic extract and hexane extract (and hexane soluble fraction of the alcoholic extract) of the aerial parts of the plant were found to demonstrate a hepatoprotective effect against liver damage in rats [1,6].

*P. niruri* has several classes of bioactive compounds such as lignans, ellagitannins, polyphenols, flavonoids, alkaloids, triterpenes, and phenyl propanoids [7]. The presence of these constituents in the plant makes it of great interest to researchers who are interested in determining its health benefits and studying the effect of processing on these metabolites. Phyllanthin and related compounds are major diaryl butane-type lignans that are present in the plant and various *Phyllanthus* species with a demonstrated broad spectrum of biological activities. Phyllanthin has a protective effect against the progression of high-fat diet-induced metabolic disturbances in mice [8], and protects primary cultured rat hepatocytes from CCl_4_- and GalN-induced cytotoxicity [9]. Phyllanthin is known to possess anti-inflammatory [10], immunomodulatory [11], nephroprotective [12], and anticancer [13] properties. It is worth mentioning that phyllanthin and hypophyllanthin transformed systemically into enterolignans that were expected to be responsible for augmenting estrus cycle in rats [14].

Considering the importance of phyllanthin and related lignans, researchers have tried to increase its extraction quality and efficiency by using various techniques. However, most of the research has been on leaves and the aerial parts of other *Phyllanthus* species; *Phyllanthus amarus* that is known to contain higher concentration of phyllanthin [15].

The conventional methods of extraction with solvents have been among the most widely used methods and researchers have found that extraction of the leaves or aerial parts with ethanol, methanol, or other organic solvents yielded extracts with a higher concentration of phyllanthin than that of aqueous extracts. Murugaiyah and Chan (2007) [16] collected the aerial parts of *P. niruri* collected from six different geographical locations in Penang, Malaysia and found that the concentration of phyllanthin in the methanolic extract was in the range of 2.37 to 7.54 mg/g extract, while total lignans were in the range of 5.53 to 17.1 mg/g. In addition, they found more phyllanthin (10.4 mg/g) in the methanolic extract of the leaves, as determined by HPLC [17].

Considering that plant cell walls and structural tissues are composed of three primary structural polymers, i.e., cellulose, hemicellulose, and lignins [18] which are a dominant form of biomass, they normally resist decomposition. Accordingly, releasing lignans from this complex matrix using solvents (or mixture of solvents) is very challenging and requires large amounts of solvent and a long extraction time [18].

Recently, nonconventional techniques have been described to disrupt or degrade plant cell walls, and thus enable better release and more efficient extraction of the bioactive compounds in a short time and with minimal solvent usage. Among these techniques, microwave-assisted extraction (MAE) of *P. amarus* leaves yielded extract with a better phyllanthin content, relative to that obtained by Soxhlet or maceration [19], and digestion with alkaline [20] or enzymes [21] was reported to optimize extraction of other types of lignans from flaxseed. Despite the importance of phyllanthin and related lignans and their contribution to the high value of *P. niruri* aerial parts used as a crude drug, little is known about the preparation of a lignan-rich extract from the plant. This study aimed to evaluate the impact of the nonconventional methods on the quality and efficiency of extracting phyllanthin and related lignans from the aerial parts of *P. niruri* [21].

## 2. Results

### 2.1. Identification of Phyllanthin-Related Lignans in the Hexane Extract of P. niruri

**Figure 1:**
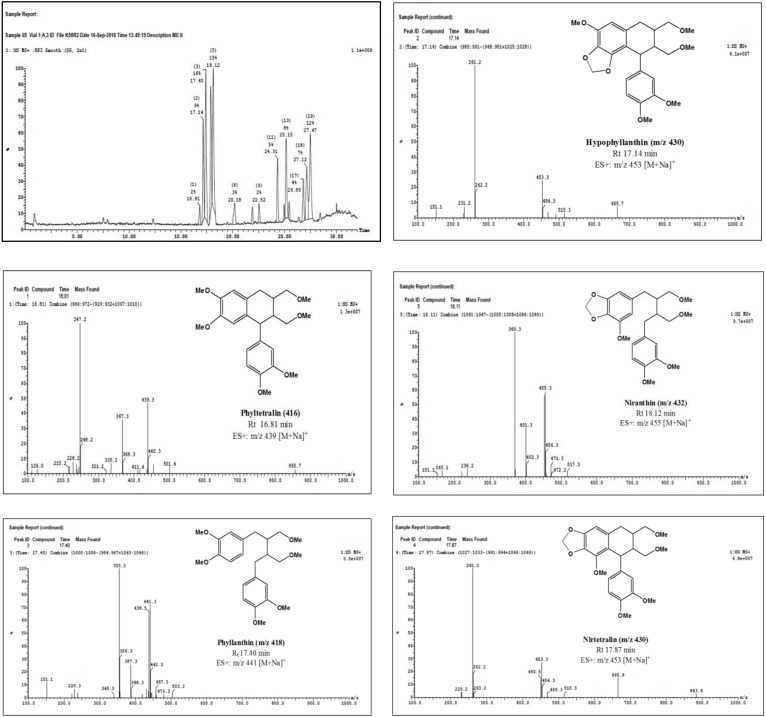
ESI-MS spectra of major lignans identified in the extract of the aerial parts of *P. niruri* L.

Tentative identification of lignans was made possible based on the comparison of the UV spectra of their clearly separated peaks with the UV spectrum of standard phyllanthin (peak 3, retention time (Rt) of 10.33 min in HPLC chromatogram). In the DAD spectra (Supplementary Materials), standard phyllanthin showed UV absorption maxima around 230 and 280 nm. Similarly, the other four peaks eluted at 8.15, 9.23, 11.61, and 13.91 min, showed UV absorption maxima around 230 and 280 nm, and were superimposed for phyllanthin-type lignans. Further confirmation of the nature of these compounds was evident by inspection of the UPLC-ESI-MS chromatograms of the five peaks (Figure 1). The peak that appeared at Rt of 17.40 min displayed a quasimolecular ion peak at *m/z* 441 [M + Na]^+^ which was typical for phyllanthin (Figure 1). Similarly, other peaks were identified as phylltetralin (Rt of 16.81 min, *m/z* 439 [M + Na]^+^), hypophyllanthin (Rt of 17.14 min, *m/z* 453 [M + Na]^+^), nirtetralin (Rt of 17.87 min, *m/z* 453 [M + Na]^+^), and niranthin (Rt of 18.12 min, *m/z* 455 [M + Na]^+^) (Figure 1).

### 2.2. Conventional Solvent Extraction

Water extract prepared from the aerial parts represents a high % yield (18.1% *w*/*w*), but HPLC analysis (Supplementary Materials) revealed the presence of phyllanthin at a very low concentration (0.33 ± 0.10 mg/g extract) (Table 1). In addition, the methanol extract was obtained at a yield of 3.6 g% *w*/*w* with a relatively low phyllanthin content (3.1 ± 2.10 mg/g). Although phyllanthin content was the highest in the hexane extract (36.20 ± 2.60 mg/g), its % yield was very low (0.82% *w*/*w*) and an almost similar content of phyllanthin (11.7 mg/g) was obtained in methylene chloride and acetone extracts with % yield of 1.12% *w*/*w* and 3.4% *w*/*w*, respectively.

### 2.3. Nonconventional Methods of Extraction

#### 2.3.1. Alkaline Digestion

Alkaline digestion of the ground plant material using KOH was superior to NaOH in enhancing the release of phyllanthin (22.34 and 11.23 mg/g extract, respectively) and total lignans (66.77 and 33.34 mg/g extract, respectively) from the alkaline digested powder when extracted with CH_2_Cl_2_ (Table 2); extracting the digested powder with a mixture of CH_2_Cl_2_-MeOH (1:1 *v*/*v*) showed lower extraction power. Moreover, exhausting the powder with boiling water prior to alkaline digestion yielded an extract with a lower content of phyllanthin and total lignans.

#### 2.3.2. Microwave-Assisted Extraction (MAE)

The MAE of the ground plant material enhanced extraction of phyllanthin, as shown in Table 3. The 80% MeOH extract obtained from the MAE showed almost a two-fold increase as compared with the MeOH extract obtained using a conventional solvent extraction method (Table 1 and Table 3). The concentration of the extract on the Diaion HP-20 column, by removing water soluble compounds, first with water, and eluting the remaining relatively nonpolar compounds using 50% MeOH, produced a lignan-rich fraction with a phyllanthin content of 21.2 mg/g extract. Moreover, the use of 50% MeOH was found to be effective in eluting almost all the lignans in its extract, as shown by the low concentration of phyllanthin (0.19 mg/g) in the subsequent acetone fraction (Table 3).

#### 2.3.3. Enzyme-Assisted Extraction (EAE)

In preliminary trials, incubation of the ground plant material with cellulase alone, and cellulase and hemicellulose or protease at different time intervals revealed that incubation with a mixture of cellulase and protease for 48 and 72 h was the most effective for better release of phyllanthin and total lignans from the ground material (data not shown). Therefore, a time course for incubation of the plant material with several mixing ratios of cellulase and protease (4U + 2U/g, 6U + 3U/g, and 9U + 4U/g, respectively) was tried, at 48 and 72 h. A substantial increase in the content of phyllanthin (25.9 mg/g extract, as 13.5 and 12.4 mg/g in the hexane and acetone extracts, respectively was obtained after incubation for 72 h with the ratio 9U and 4U/g (Table 4). On the one hand, a high content of total lignans (85.87 mg/g extract, calculated as phyllanthin equivalent) was obtained from both *n*-hexane and acetone extracts (Table 4). On the other hand, the % yield of the extracts was optimized (13.92% *w*/*w*, as 4.50 and 9.42% *w*/*w* in hexane and acetone extract, respectively). However, other enzyme combinations showed extracts with a lower % yield (3.71% to 5.1% *w*/*w*) with average contents of phyllanthin (around 6.0 mg/g extract) and total lignans (up to 21.00 mg/g extract) (Table 4).

Although the enzyme treatments did not attain the highest phyllanthin content obtained through direct extraction of the powder using *n*-hexane (Table 1), the % yield of the resulting extracts were five- to 10-fold higher (9.4% and 4.5%) than that of the *n*-hexane extract (0.8% *w*/*w*).

The results obtained, in this study, showed that the cell membrane of the aerial parts of *P. niruri* was efficiently disrupted after incubation with some of the hydrolytic enzymes such as protease, and cellulase after incubation for 72 h. The combination of the two enzymes, protease and cellulase with specific units and ratios, effectively broke down the cell membrane and facilitated the extraction of phyllanthin and related lignans. The amount of phyllanthin reached up to 13.5 mg/g in the *n*-hexane extract of the aqueous media and up to 12.4 mg/g in the acetone extract of the enzymes-digested marc (Table 4), and the total amount of lignans increased up to 85.78 mg/g extract (Table 4).

## 3. Conclusions

**Figure 2:**
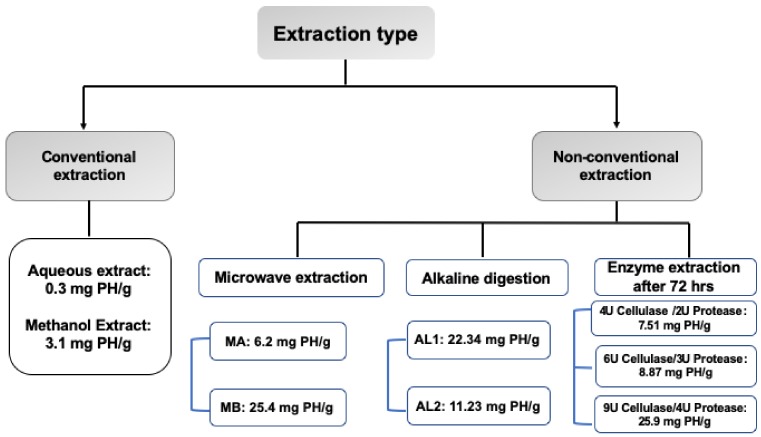
Diagrammatic presentation for phyllanthin yield by conventional vs. nonconventional methods. PH, phyllanthin; MA, the 80% aqueous MeOH extract; MB, the dry extract obtained after eluting MA from the Diaion HP-20 column using 50% MeOH; AL1, CH_2_Cl_2_ extract of KOH-digested powder; AL2, CH_2_Cl_2_ extract of NaOH-digested powder.

Currently, there are several well-established methods for the extraction of natural products from various available sources. The choice of an appropriate extraction protocol depends on the overall target of the extraction process, the nature of the source material, and the target compounds. The aim of this study was to prepare an extract rich in phyllanthin and related lignans from *P. niruri* aerial parts Figure 2. These compounds are of similar and moderate polarity and are difficult to extract using polar solvents such as water, EtOH, and MeOH. However, extraction with selective nonpolar solvents such as *n*-hexane, methylene chloride, and acetone produced extracts with poor yield and relatively better content of lignans.

The above findings suggest that the solvent extraction process itself is unlikely to be able to produce an extract with a high yield. Therefore, three nonconventional protocols were followed to produce extract with a high yield and rich in phyllanthin and related lignans. Relative to solvent extraction methods (Table 1), the yield and content of lignans were significantly optimized after adopting MAE or digesting the ground plant material with KOH or enzyme combination before extraction with nonpolar solvents (Figure 2, Table 2, Table 3 and Table 4).

## 4. Materials and Methods

### 4.1. Chemicals and Reagents

Solvents of HPLC grade were used for HPLC analysis, and the analytical grade solvents for extraction and thin layer chromatography (TLC) were purchased from Sigma-Adrich (St. Louis, MO, USA). Phyllanthin (Fluka Lot # BCBL2476V, product of India), cellulose, hemicellulose, and protease were purchased from (Sigma Chemical Co. Japan, Shinagawa, Japan).

### 4.2. Plant Material

The aerial parts of *P. niruri* were collected from Tasek Gelugor, Penang, Malaysia and identified by a staff member of the Malaysian Agricultural Research and Development Institute (MARDI) and a voucher specimen (PN-01-082018) was kept at the herbarium of the Faculty of Pharmacy, Cairo University.

### 4.3. Conventional Extraction

Samples of the ground plant material (100 g) were separately extracted with boiling water and methanol (2 × 500 mL × 5 min) using a sonication bath (Trassonic TS 540, Singen, Germany). After filtration, the aqueous filtrate was freeze-dried to give 18.10 g of dry residue (aqueous extract), while the methanol extract was evaporated under reduced pressure to give 3.6 g of soft residue (methanol extract). Similarly, samples (100 g) were separately extracted with 1 L of *n*-hexane, CH_2_CL_2_, and acetone using Soxhlet to yield 0.8 g, 1.1 g, and 3.4 g of dry residues, respectively. The yield of extract and phyllanthin content were determined and a known amount of the extract was dissolved in methanol to give a concentration of 10 mg/mL, filtered through 0.45 um syringe filters, and kept in the refrigerator until analysis by HPLC (results are shown in Table 1).

### 4.4. Nonconventional Methods for Extraction of Lignans

#### 4.4.1. Alkaline Digestion

Samples (10 g each) of the ground plant material were separately mixed with 30% aqueous solution of KOH or NaOH (25 mL) and left to dry at room temperature (>24 h). The powder after alkaline digestion was extracted with CH_2_Cl_2_ (3 × 100 mL, 5 min each) using a sonication bath, and filtered. The filtrate was evaporated to yield (AL1 and AL2) for KOH and NaOH digested extracts, respectively. The extraction yield and phyllanthin content were determined and the results are shown in Table 2.

#### 4.4.2. Microwave-Assisted Extraction (MAE)

The MAE was performed with an open system microwave apparatus, Mars CEM 240/50, model number 907511, serial number MD 3728. A sample of the ground material (15 g) was impregnated with water, pressed, and the wet powder was transferred to a 100 mL 4-necked round bottom flask. Then, 100 mL of 80% (*v*/*v*) methanol-water was added, mixed well, and the flask was irradiated at a power of 600 W, and the temperature was set between 23 and 67 °C. After the extraction was completed, the flask was removed from the oven and the contents filtered. Then, the residue was rinsed with 80% MeOH (2 × 100 mL) and filtered. The filtrate was collected and concentrated under reduced pressure to give extract MA (1.22 g). MA was applied to a column of the Diaion HP-20 (10 × 1.0 cm) and eluted first with 50% aqueous MeOH (3 × 100 mL) which was collected and evaporated to give MB (0.92 g), and then eluted with acetone (3 × 100 mL) which upon evaporation gave MC (0.23 g). Phyllanthin content and percentage yield of MA and its fractions, MB and MC, were determined (see Table 3) [19]. It has been reported that preliminary impregnation of matrices with water is beneficial for high recovery of active constituents by microwave-assisted extraction. Using 80% MeOH and extraction for 3 min was reported to be ideal for better extraction of phyllanthin from *Phyllanthus* species [19].

#### 4.4.3. Enzyme-Assisted Extraction

##### Screening of Different Enzymes and Enzymes Combinations

According to preliminary trials (data not shown), the experimental parameters, i.e., enzymes selected (cellulase, hemicellulase, and protease), their combination, and ratio, were investigated. All the samples were prepared and analyzed in triplicate. Briefly, samples of ground material pre-extracted with boiling water (5 g each) were transferred into conical flasks (250 mL capacity) and distilled water (100 mL) was added. The content was mixed well, and the pH of the suspension was adjusted to the appropriate pH according to the enzyme used. To each flask, cellulase (2 U/g), mixtures of cellulase (2 U/g), and protease (1 U/g), or cellulase (2 U/g) and hemicellulase (1 U/g), were separately added (for mixture enzymes, cellulase was added first and the other enzyme was added 12 h later), and the flasks were incubated at 50 °C in a water bath. Then, the flasks were removed at time intervals, 24, 48, and 72 h, and worked out to extract and analyze the phyllanthin content and related lignans in the solution and powder, as shown hereunder. The content of each flask was separately filtered using Whatman filter papers to give the filtrate and marc parts. The filtrate was extracted with hexane (3 × 50 mL) and the hexane layer was collected and evaporated to dryness under reduced pressure to give a dry residue “Hexane Fr.”. The marc part was extracted thrice with acetone (3 × 50 mL, 5 min each) using a sonication bath, and filtered. The acetone extracts were collected and evaporated to give “Acetone Fr.” and the content of phyllanthin and related lignans (calculated as mg phyllanthin equivelant/g fraction) in each fraction was determined by HPLC.

##### Optimization of Enzymatic Reactions

On the basis of the screening results above (data not shown), a combination of cellulase and protease enzymes was found to effectively enhance the release of lignans from the ground plant material. Accordingly, the time course for the effect of different ratios (4:2 U/g, 6:3 U/g, and 9:4 U/g, respectively) of cellulase and protease on the extraction of lignans was investigated. Similarly, a blank experiment (without enzyme) was carried out for comparison. The aqueous solution was partitioned with *n*-hexane (3 × 50 mL) and the *n*-hexane layer was evaporated under reduced pressure to give “Hexane Fr.”. The marc was extracted thrice with acetone (3 × 50 mL, 5 min each) using an ultrasonic bath, and filtered. The acetone extracts were collected and evaporated to give “Acetone Fr.”, the extraction yield and content of phyllanthin and related lignans in each fraction were determined, and the results are compiled in Table 4.

#### 4.4.4. HPLC Analysis of Lignans

The quantification of phyllanthin and related lignans in *P. niruri* aerial parts was performed with an HPLC instrument (Agilent HP1200 series, Santa Clara, USA), equipped with a G1322A quaternary pump, a degasser, and a photodiode array detector (PDA). Chromatographic separation was achieved on an Agilent ZORBAX Eclipse XDB-C18 (150 × 4.6 mm i.d., 5 μm), including a C-18 guard column. For gradient elution, mobile phase A and B were employed; A was 0.1% TFA in water and B was methanol. The following gradient was used: Isocratic at 70% B for 16 min, then to 100% B in 2 min, and kept at 100% B for 5 min. Peaks were monitored at 230, 280, and 320 nm. The flow rate was maintained at 1.0 mL/min prior to HPLC analysis; all samples were filtered using Millex-HV filters (Millipore, Bedford, MA, USA) with 0.45 µm pore size. Injection volume was 20 μL. Phyllanthin was used as an external standard. A standard calibration curve of phyllanthin showed linearity within dilutions ranging from 37 to 500 µg/mL with an LOD of 40 ug/mL and LOQ of 133 ug/mL (Supplementary Materials). Using the above conditions, phyllanthin was eluted at 10.33 min. The concentrations of phyllanthin and related lignans (expressed as mg phyllanthin equivalent) in the extract were calculated from the calibration curve of phyllanthin (see Supplementary Materials).

#### 4.4.5. UPLC/ESI-MS Analysis of Lignans

UPLC-ESI-MS analysis of the extract was carried out to tentatively identify peaks corresponding to phyllanthin and related lignans.

##### Instrument

A XEVO TQD triple quadruple instrument (Waters Corporation, Milford, MA01757 U.S.A, mass spectrometer) was used and column, ACQUITY UPLC-BEH C18, 1.7 µm, 2.1 × 50 mm, flow rate 200 mL/min. The mobile phase was composed of water containing 0.1% formic acid in water (Solvent A) and 0.1% formic acid in methanol (Solvent B) by applying the following mobile phase gradient: 10% B (0.0 to 2 min), 10% to 30% B (in 3.0 min), 30% to 70% B (in 10.0 min), 70% to 90% B (for 3.0 min), and 90% to 100% B (in 1.0 min). Liquid chromatography separation and mass spectrometric detection were achieved by employing ESI-MS.

##### Procedure for UPLC-ESI-MS Analysis

Part of the extract was dissolved in HPLC grade methanol to give (100 μg/mL) solution. The solution was filtered using a membrane disc filter (0.2 μm), degassed by sonication before injection and the sample (10 μL) was subjected to UPLC-ESI-MS analysis. The parameters for analysis were carried out using positive ion mode as follows: Source temperature 150 °C, cone voltage 30 eV, capillary voltage 3 kV, desolvation gas temperature 440 °C, cone gas flow 50 L/h, and desolvation gas flow 900 L/h. The eluted compounds were identified in the ESI at mass spectra ranging from 100 to 1000 m/z. The peaks and spectra were processed using Mass Lynx 4.1 software (Waters) and tentatively identified by comparing retention time (Rt) and mass spectrum with reported data.

#### 4.4.6. Data Analysis

Data were expressed as means ± standard deviations of the means of three replicated determinations.

## Figures and Tables

**Table 1:** Contents of phyllanthin and total lignans in extracts of *P. niruri* aerial parts, prepared by conventional methods.

Conventional Extract	Phyllanthin Content (mg/g Extract)	Total Lignans Content (calc. as Phyllanthin mg/g Extract)	Yield of Extract (g% *w*/*w*)
Water extract	0.33 ± 0.10	2.14 ± 0.81	18.10
Methanol extract	3.10 ± 2.10	15.15 ± 2.10	3.60
Hexane extract	36.20 ± 2.60	115.1 ± 8.30	0.82
CH_2_Cl_2_ extract	11.70 ± 1.68	41.55 ± 1.32	1.12
Acetone extract	11.70 ± 1.10	38.99 ± 4.20	3.40

**Table 2:** Effect of different alkaline digestion conditions on phyllanthin and total lignan contents.

	Extraction Method *	Phyllanthin (mg/g extract)	Total Lignans (calc. as Phyllanthin mg/g Extract)	Yield of Extract (g% *w*/*w*)
**Ground Plant Material**	AL1	22.34 ± 0.13	66.77 ± 0.67	3.1
	AL2	11.23 ± 0.35	33.34 ± 1.17	4.9

* AL1, CH_2_Cl_2_ extract of KOH-digested powder; AL2, CH_2_Cl_2_ extract of NaOH-digested powder.

**Table 3:** Contents of phyllanthin and total lignans in the methanol extract and fractions of *P. niruri* using microwave-assisted extraction (MAE).

Microwave Extraction	Phyllanthin (mg/g Extract)	Total Lignans (calc. as Phyllanthin mg/g Extract)	Yield of Extract (g% *w*/*w*)
**MA**	6.20 ± 1.05	20.6 ± 1.9	8.13 *
**MB**	25.4 ± 1.30	64.20 ± 4.2	75.0 **
**MC**	0.23 ± 0.82	2.07 ± 0.50	19.0 **

*, % yield from powder; **, %yield from MA; MA, the 80% aqueous MeOH extract (1.22 g) from MAE of 15 g of ground plant material; MB, the dry extract (0.92 g of MA) obtained after eluting MA from the Diaion HP-20 column using 50% MeOH; MC, the extract (0.23 g of MA) obtained after successive elution of MA from the Diaion HP-20 column with acetone.

**Table 4:** The effect of different concentrations and incubation times of cellulase and protease on phyllanthin and total lignans content.

Treatment	Incubation Time (h)	Fraction	Phyllanthin Content (mg/g Extract)	Total Lignans (calc. as Phyllanthin mg/g Extract)	Yield of Extract (g%*w*/*w*)
(C-4U/g + P-2U/g)	48 h	Hexane	0.49 ± 0.23	1.04 ± 1.03	1.32
		Acetone	3.02 ± 0.51	6.83 ± 1.23	4.01
(C-6U/g + P-3U/g)		Hexane	0.42 ± 0.10	3.45 ± 0.92	1.40
		Acetone	5.32 ± 0.33	12.37 ± 1.50	3.57
(C-9U/g + P-4U/g)		Hexane	1.09 ± 1.02	4.67 ± 0.98	1.21
		Acetone	6.47 ± 0.82	14.53 ± 0.85	4.23
(C-4U/g + P-2U/g)	72 h	Hexane	1.21 ± 0.32	4.72 ± 1.24	2.31
		Acetone	6.30 ± 0.90	18.92 ± 2.10	5.10
(C-6U/g + P-3U/g)		Hexane	2.70 ± 0.12	8.66 ± 1.53	1.90
		Acetone	6.17 ± 0.28	21.05 ± 1.21	3.71
(C-9U/g + P-4U/g)		Hexane	13.50 ± 1.29	50.67 ± 1.57	4.50
		Acetone	12.40 ± 1.78	35.20 ± 1.86	9.42
Blank		Hexane	0.60 ± 0.43	-	2.20
		Acetone	2.90 ± 0.52	-	6.10

C, cellulase enzyme; P, protease enzyme.

## Supplementary Materials

The following are available online at https://www.mdpi.com/1420-3049/25/5/1179/s1, Figure S1: HPLC profiles for lignans-enriched fraction, Figure 2: UV spectra (DAD) of lignans identified in the lignans-enriched extract of the aerial parts of P. niruri, Figure 3: standard calibration curve of phyllanthin.
