# Supplementary material for: Preparation of Lignan-Rich Extract from the Aerial Parts of Phyllanthus niruri Using Nonconventional Methods
Source: Molecules. 2020 Mar 5;25(5):1179. doi: 10.3390/molecules25051179 (PMC7179407; doi:10.3390/molecules25051179)
Supplement: Supplementary file 1 [file molecules-25-01179-s001.pdf]

Supplementary materials

Preparation of Lignan-Rich Extract from the Aerial Parts of *Phyllanthus niruri* Using Nonconventional Methods

Meselhy R. Meselhy [1], Ola E. Abdel-Sattar [1], Sahar El-Mekkawy [2], Ahmed M. EL-Desoky [3], Shanaz O. Mohamed [4], Sobhy M. Mohsen [5], Essam Abdel-Sattar [1], Ali El-Halawany [1,*]

[1] Department of Pharmacognosy, Faculty of Pharmacy, Cairo University, Kasr El Aini st., 11562 Cairo, Egypt; meselhy.meselhy@pharma.cu.edu.eg (M.R.M.); ola.abdelsattar@gmail.com (O.E.A.-S.); essam.abdelsattar@pharma.cu.edu.eg (O.E.A.-S.)

[2] Department of Chemistry of Natural Compounds, National Research Centre, 12622 Dokki, Egypt; saheg.2011@gmail.com

[3] Department of Molecular Biology, Genetic Engineering and Biotechnology Research Institute (GEBRI), University of Sadat City (USC), 32958 Sadat City, Egypt; ahmed.desoky334@gmail.com

[4] School of Pharmaceutical Sciences, Universiti Sains Malaysia, 11700 Gelugor, Penang, Malaysia; shahnas@mynaturalwellness.com

[5] Department of Food Science and Technology, Faculty of Agriculture, Cairo University, 12613 Giza, Egypt; sobmohsen1@hotmail.com

* Correspondence: ali.elhalawany@pharma.cu.edu.eg; Tel.: +202-25353100; Fax: +202-23628246

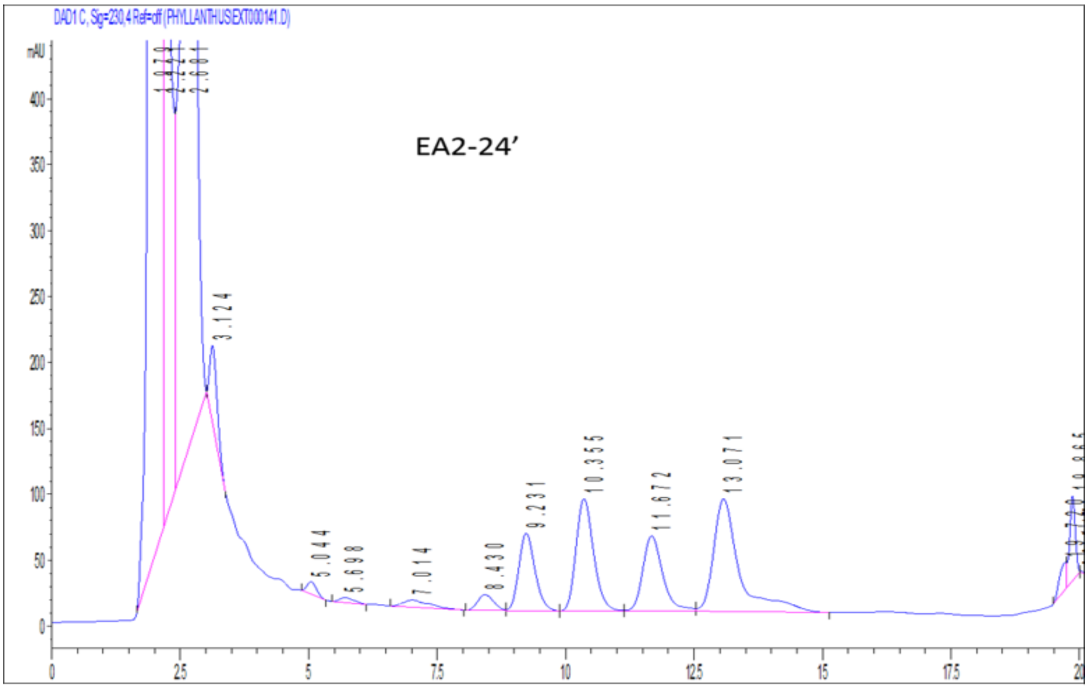

Figure S1. HPLC profiles for lignans-enriched fraction.

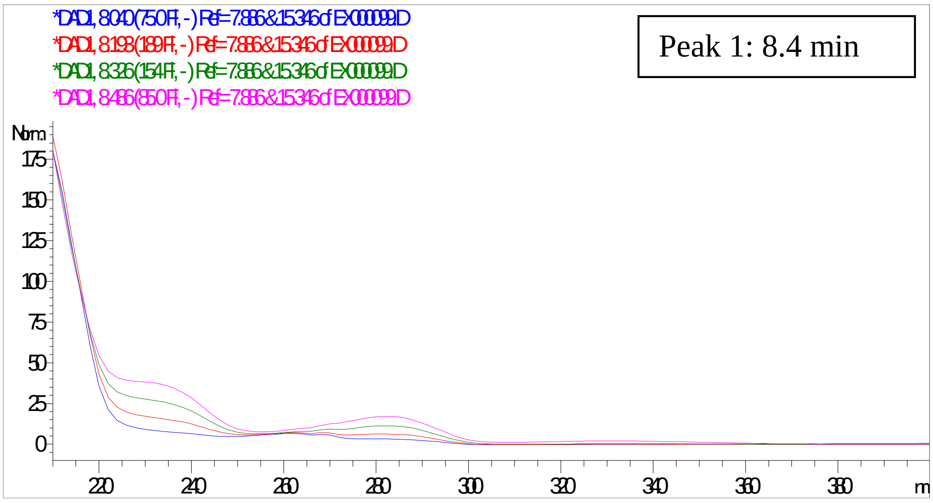
Peak 1: 8.4 min
Norm.
175
150
125
100
75
50
25
0
220
240
260
280
300
320
340
360
380
nm

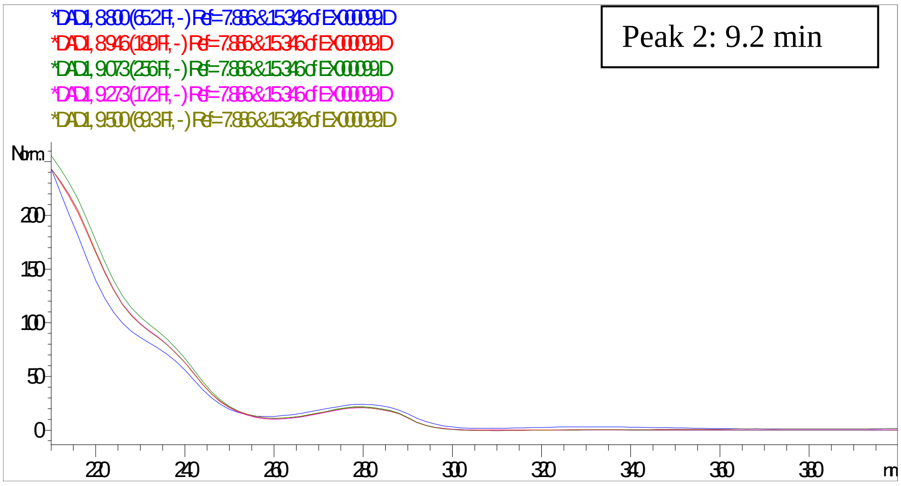
Peak 2: 9.2 min
Norm.
200
150
100
50
0
220
240
260
280
300
320
340
360
380
nm

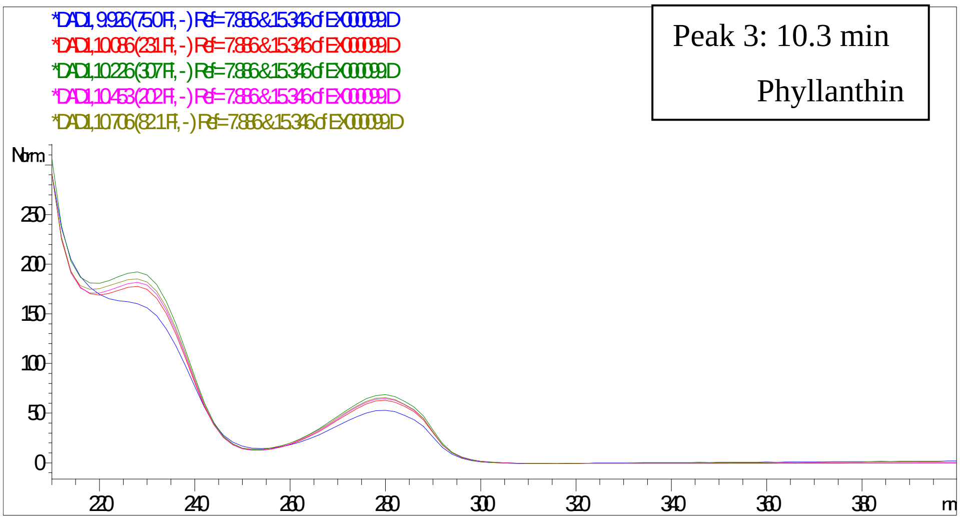
Peak 3: 10.3 min
Phyllanthin
Norm.
250
200
150
100
50
0
220
240
260
280
300
320
340
360
380
nm

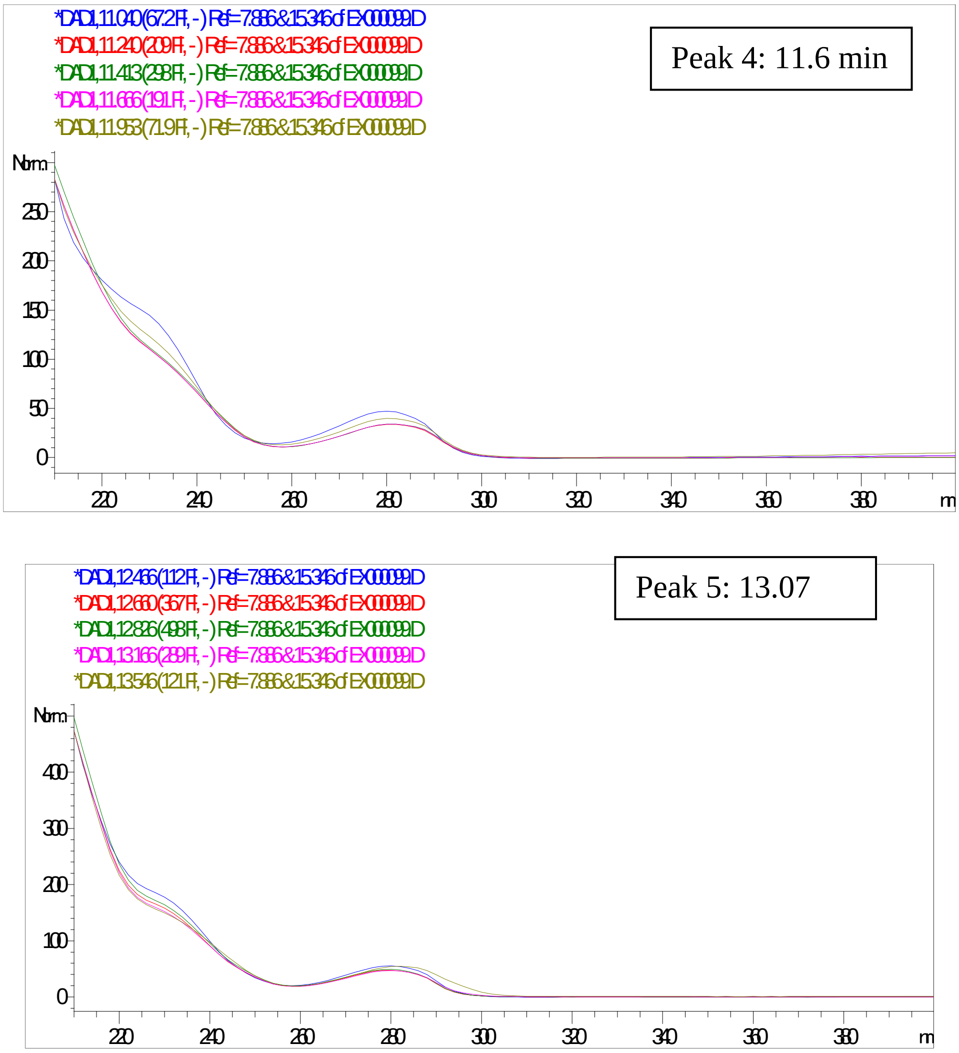

Figure S2. UV spectra (DAD) of lignans identified in the lignans-enriched extract of the aerial parts of *P. niruri.*

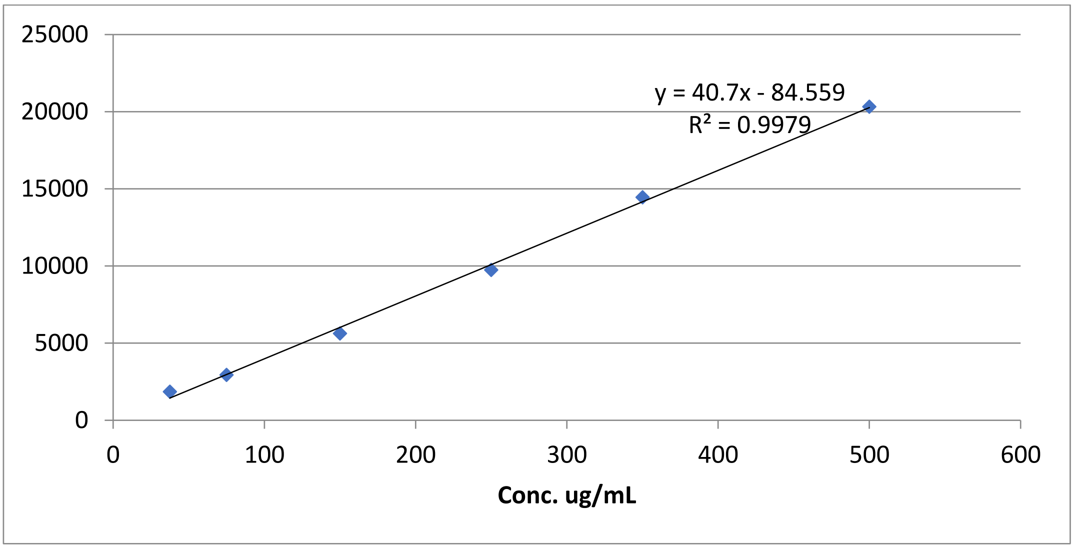

Figure S3. standard calibration curve of phyllanthin.
